# Prevalence and Clinical Features of Celiac Disease in a Cohort of Italian Children with Autism Spectrum Disorders

**DOI:** 10.3390/nu13093046

**Published:** 2021-08-30

**Authors:** Margherita Prosperi, Elisa Santocchi, Elena Brunori, Angela Cosenza, Raffaella Tancredi, Filippo Muratori, Sara Calderoni

**Affiliations:** 1 Department of Developmental Neuroscience, IRCCS Fondazione Stella Maris, 56128 Pisa, Italy; margherita.prosperi@fsm.unipi.it (M.P.); elisa.santocchi@fsm.unipi.it (E.S.); elena.brunori@fsm.unipi.it (E.B.); angela.cosenza@fsm.unipi.it (A.C.); raffaella.tancredi@fsm.unipi.it (R.T.); filippo.muratori@fsm.unipi.it (F.M.); 2 Department of Clinical and Experimental Medicine, University of Pisa, 56126 Pisa, Italy

**Keywords:** autism spectrum disorders, children, celiac disease, gastrointestinal symptoms

## Abstract

Background: Autism spectrum disorders (ASD) are a heterogeneous group of neurodevelopmental conditions whose etiopathogenesis derives from a complex interaction between genetic liability and environmental factors. In this framework, mounting evidence suggests that immune system dysfunction could be a risk factor contributing to the development of ASD in at least a subpopulation of individuals. In particular, some studies suggest an association between celiac disease (CD)—a long-term autoimmune disorder that primarily affects the small intestine triggered by the ingestion of gluten—and ASD, while others hypothesized a random link. This investigation aimed to evaluate the prevalence of CD in a large sample of school-aged children with ASD and to characterize their clinical profile. Methods: Medical records of 405 children with ASD aged 5–11 years (mean age: 7.2 years; SD: 1.8 years) consecutively referred to a tertiary-care university hospital between January 2014 and December 2018 were reviewed; among them, 362 had carried out serological testing for CD. Results: Nine patients with positive CD serology were identified, eight of which satisfied the criteria for CD diagnosis. The estimated CD prevalence in ASD children was 2.18% (95% CI, 0.8–3.7), which was not statistically different (1.58%; *p* = 0.36) from that of an Italian population, matched for age range, considered as a control group (95% CI, 1.26–1.90). Three out of the eight ASD patients with CD did not have any symptoms suggestive of CD. Conclusions: Our findings did not show a higher prevalence of CD in ASD children than in the control population, but could suggest the utility of routine CD screening, given its frequent atypical clinical presentation in this population.

## 1. Introduction

Autism spectrum disorders (ASD) are neurodevelopmental disorders characterized by persistent social communication difficulties with concurrent restricted interests, repetitive activities, and sensory abnormalities [1]. Although it is well-known that ASD derives from a complex interplay between genetic predisposition and environmental risk factors [2], etiology is still largely unknown. Recent studies, both in animal models and humans, detected an immune system dysregulation in ASD [3], supporting the involvement of an altered immunity system in the pathogenesis of these conditions. Moreover, a family history of autoimmune diseases [4] and altered immune responses [3] have been associated with symptoms of ASD. Besides, allergies and autoimmunity diseases appeared significantly more common in children with ASD than in matched controls, with an odds ratio of 1.22 and 1.36, respectively [5]. In addition, the similarity of specific major histocompatibility complex (MHC) haplotypes and polymorphisms has been detected in genes related to self-tolerance/immune regulation and ASD [6].

Among diseases with autoimmune pathogenesis, conflicting results emerge regarding an increased prevalence of celiac disease (CD) in subjects with ASD [7]. CD is a chronic, immunomediated, systemic disease precipitated by exposure to dietary gluten in genetically susceptible individuals, with a different range of clinical manifestations [8]. Indeed, symptoms of CD vary from typical gastrointestinal (GI) problems to an extra-intestinal involvement, including iron-deficiency anemia and ataxia [9]. The clinical presentation of CD in the pediatric population is often characterized by poor growth, diarrhea, fatigability [10,11]. In addition to the classic form of CD, other presentations of the disorder are also described: (i) the “silent” form, i.e., an asymptomatic form of CD; (ii) the “potential” form, in which a positive CD-serology along with a genetic predisposition to CD (positivity of the locus HLA-DQ2 and/or -DQ8) are present without the typical alterations of the small-bowel mucosa [9,12]; (iii) the “atypical” form, characterized by few or no gastrointestinal symptoms, and a variety of extra-intestinal manifestations, including neurologic, dermatologic, hematologic, endocrinologic, reproductive, renal, psychiatric, skeletal and liver involvement [9].

CD’s overall prevalence is close to 1% in Western populations and about 1.15% in Italy, based on unselected population serological screenings [13]. The prevalence of CD in children has significantly increased in the last decades: indeed, in the nineties, the prevalence was 0.54% in the general school-age pediatric population [14,15], whereas a recent investigation identified a prevalence of more than 1.5% [16]. Of note, if the increase in CD prevalence over time is a “true” increase in cases or a function of a greater awareness and improvement in screening and diagnostic methods is not entirely understood. Furthermore, selected pediatric populations, such as subjects with Down syndrome or with other chromosomal syndromes, have a higher risk for CD than the general population, and systematic screening for CD is recommended in managing these individuals [17,18].

Regarding ASD, the American Academy of Pediatrics recommends that every ASD child with GI symptoms should be evaluated with specific exams testing the GI tract [19]. Pediatricians often have difficulty recognizing signs of GI problems [20] and completing a medical examination that includes an accurate evaluation and follow-up of corporeal and *weight*-*for*-*height* development because of the frequent difficulty in visiting children with ASD and communication barriers typical of these patients. For these reasons, it could be very useful in the ASD population for the first step of screening for CD to be a blood sample analysis.

To date, some studies have detected an association between CD and ASD [21,22,23,24,25]. Other research did not find a relationship between these two conditions [7,26,27,28,29,30,31,32,33,34,35], hypothesizing a random link. Some authors have suggested the presence of a subgroup of ASD patients with increased immune reactivity to gluten, different from that of a typical CD [7,33,34]. However, there is little evidence that a gluten-free diet (GFD) could positively affect ASD symptoms in patients without CD comorbidity [36]. For all these reasons, it seems crucial to better understand whether an association between autism and CD exists.

It is worth noting that a substantial portion of published studies investigating the prevalence of CD in ASD subjects examined limited samples (less than 100 subjects per study), with a wide range of ages and without the exclusion of ASD subjects already on GFD (possible false-negative results). Moreover, diagnosis of CD is heterogeneous among studies and sometimes inaccurate, e.g., by using different criteria to define CD or in the absence of an intestinal biopsy after positive CD serological testing [37]. For decades, anti-gliadin antibodies (AGA), which are thought to have low specificity for CD, were used in the first screening for CD. In the last decade, thanks also to recently published ESPGHAN guidelines and the widespread use of transglutaminase (tTG) and endomysial autoantibodies (EMA), the serological screening became more specific for CD [38].

Starting from the abovementioned controversial and inconclusive literature, this study aims to evaluate the prevalence of CD in a large sample of children with ASD, aged from 5 to 11 years, compared with a pediatric population matched for age range.

## 2. Methods

### 2.1. Participants

We retrospectively reviewed data of all inpatient and day-patient children referred between January 2014 and December 2018 for a suspected diagnosis of ASD at a tertiary care university hospital (N = 1424). At the end of the diagnostic evaluation, 1234 subjects received a diagnosis of idiopathic ASD according to DSM-5 criteria [1]. ASD diagnosis was performed by a multidisciplinary team (a senior child psychiatrist, an experienced clinically trained research child psychologist, an educational therapist, and a speech-language pathologist) during 5–7 days of extensive evaluation, including the Autism Diagnostic Observation Schedule-Second Edition (ADOS-2) [39,40].

These subjects were consecutively examined, and all the patients with (a) neurological syndromes or focal neurological signs; (b) significant sensory impairment (e.g., blindness, deafness); (c) potential secondary causes of ASD revealed by array comparative genomic hybridization (a-CGH), DNA analysis of Fragile-X, or screening tests for inborn errors of metabolism were excluded.

**Figure 1:**
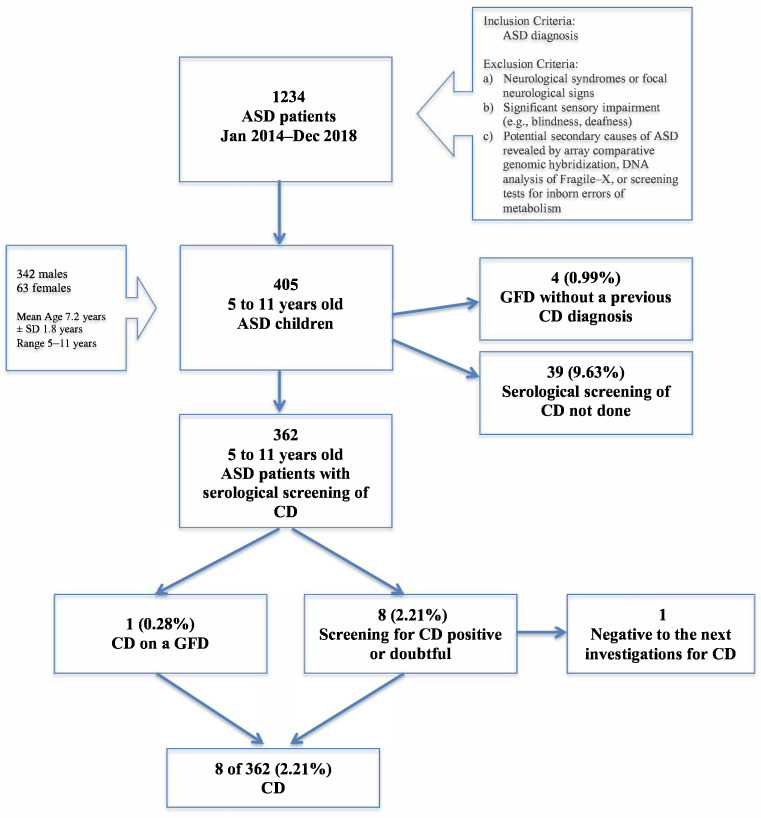
Study flow-chart. Abbreviations (alphabetic order): ASD, Autism Spectrum Disorders; CD, celiac disease; GFD, gluten-free diet; SD standard deviation.

From an initial sample of 1234 ASD children, we selected the subgroup of subjects aged 5 to 11 years (N = 405, see Figure 1). This choice was motivated by the possibility of comparing the CD prevalence obtained in the current study with the CD prevalence recorded in a random sample of children from the community, matched for age range and geographical origin [16].

### 2.2. Celiac Disease Testing and Diagnosis

During the first evaluation at the ASD unit of our institute, all children usually undergo a serological testing for CD, as part of their assessment, by determining the titers of anti-tTG immunoglobulin (Ig)A and IgG and their total serum IgA concentrations. Anti-endomysium (EMA) IgA antibodies are also assayed. We are aware that EMA determinations are considered confirmatory tests performed only in children with positive anti-tTG IgA levels and should not be intended as first-line screening tests for CD [41]. However, we decided to take full advantage of the unique blood draw we could perform in our hospital (most ASD patients do not make a follow-up evaluation). In this way, we limited the number of subsequent blood draws in case of anti-tTG positivity in this population, which is particularly vulnerable and stressed by medical procedures.

Anti-transglutaminase antibodies (anti-tTG) are quantified with fully automated EliA™ Celikey^®^ on ImmunoCAP 250 (Phadia -Uppsala, Sweden-) and measured with fluorescent enzyme immunoassay (FEIA). The upper limit of their physiological range is 10 U/mL values; between 7 and 10 U/mL are considered “borderline”; values under 7 U/mL are considered negative. EMA IgA is measured with indirect immunofluorescence on Euroimmun slides, and it is subject to significant variability in the interpretation of results. Still, its specificity is very high (98–100%) [42]. Possible EMA results in our sample were: “+”: positive result; “−”: negative result; “+/−”: doubt/borderline result. Total serum IgA concentrations were determined using the nephelometric technique and compared with cutoff values for age.

CD serological results of each ASD subject were pulled from manual, retrospective chart reviews of the electronic medical record. The CD serological test was considered positive when the anti-tTG IgA antibodies (Ab) titer was >10 U/mL and was confirmed by a positive EMA-IgA test. In cases of IgA concentration deficiency, we considered an IgG-based test.

In children with positive CD serological results, we systematically checked standardized call interviews for the presence of symptoms suggestive for CD, and for whether a pediatric gastroenterologist had subsequently confirmed the CD diagnosis. To this aim, we asked parents to send us, by e-mail, the reports of the exams performed and a copy of the CD illness certificate, if present. Moreover, a standardized telephone interview with parents, specially created for this study, was also conducted, during which we asked whether the classic (abdominal distension, anorexia, chronic or recurrent diarrhea, failure to thrive or weight loss, irritability, muscle wasting) and non-classic manifestations (arthritis, aphthous stomatitis, constipation, dental enamel defects, dermatitis herpetiformis, hepatitis, iron-deficient anemia, pubertal delay, recurrent abdominal pain, short stature, vomiting) most frequently reported in association with CD in pediatric populations [10] were present in the child before the CD screening, and their familiarity with CD.

As per the ESPGHAN guidelines [43,44], we referred to the following diagnostic confirmation criteria to define a diagnosis of CD: (i) a combination of at least one positive celiac-specific serologic test, such as anti-tTG IgA (or IgG in cases of IgA deficiency) and EMA, and demonstration of histologic changes of modified Marsh grade 2 or more on the small intestinal biopsies; and (ii) high anti-tTG levels (>10 times ULN) and positivity of EMA in the presence of HLA-DQ2/8 in symptomatic children, without a duodenal biopsy.

The CD prevalence in the eligible population was calculated considering: (i) the same prevalence of CD among children who did not perform the CD testing; and (ii) the inclusion of the cases of known CD prior to testing, in line with the methodology of the study from which we assumed the CD control prevalence [16].

### 2.3. Statistical Analysis

Statistical calculations were performed with SPSS^®^ version 19.0 (Chicago, IL, USA). We performed the Chi-square test to compare the prevalence of CD between our sample and the matched Italian population [16]. A *p*-value < 0.05 was considered statistically significant.

## 3. Results

The initial sample of ASD children consisted of 405 children, 342 males (84.44%) and 63 females (15.56%), with an age range between five and eleven years (mean age: 7.2 years; SD: 1.8 years). Forty-three subjects did not screen for CD due to a concurring gluten-free diet (GFD) without a CD diagnosis (4 subjects), or to lack of an adequate blood sample (39 subjects) (e.g., hemolyzed specimen, refusal by parents to allow a blood draw). Therefore, the final sample consisted of 362 school-aged children with a median age of 6.6 years, who had performed the CD serological testing or were already in GFD for a previous CD diagnosis (only one child). Males and females were represented in a different percentage in the total sample (83.43% vs. 16.57% respectively, i.e., 302 males and 60 females), with a male-to-female ASD ratio similar to that reported in the literature (5:1) [45].

The retrospective review of their medical records identified nine patients with CD or with a positive CD serology (Table 1), namely five females and four males, with a mean age of 7.7 years (SD: 2.2; range: 5.8–11.6).

Based on the patient’s medical records and information derived from call interviews to parents, one out of these nine subjects was found to have already received a CD diagnosis and was on a GFD at the time of her first neuropsychiatric evaluation in our institute. Therefore, CD serology was not repeated in this case (child No. 4, Table 1). She had had CD serological screening performed at 18 months due to poor weight gain after the first year of life. Her CD serological screening tested positive, and multiple duodenal biopsies confirmed her CD diagnosis.

The other eight patients with positive CD serology were subject to further evaluation with a pediatric gastroenterologist, after the neuropsychiatric evaluation in our institute: Multiple duodenal biopsies were performed on four patients (children No. 2, 5, 7 and 8) confirming their CD diagnoses; in two children (children No. 1 and 3), serological screening was sufficient for their CD diagnoses (anti-tTG levels > 10 times ULN, and EMA in the presence of HLA-DQ2/8); CD screening was repeated for one patient (child No. 6), and their positivity was confirmed, but her parents refused to allow a biopsy. One subject was negative to subsequent investigations for CD (child No. 9).

Overall, eight out of 362 children satisfied the CD diagnostic criteria, and seven of them had full-blown CD. None had a known family history of CD, and only one had previously been screened for CD (see Table 1).

Three out of the eight patients (children No. 1, 2 and 8) did not have symptoms suggestive of CD. In the remaining five patients, some signs/symptoms suggestive of CD are reported: specifically, growth delay with poor weight gain in two (children No. 3 and 4), gastrointestinal symptoms (severe and mild constipation, respectively) associated with neurovegetative symptoms (insomnia/irritability and insomnia, respectively) in two (children No. 5 and 6), and non-specific dermatological manifestations in child No. 7.

The estimated CD prevalence in the eligible study group was calculated assuming: (i) the same prevalence of CD diagnoses (7/362; 1.93%) among the 43 children on whom screening was not performed (representing an additional 0.83 estimated cases of CD), and (ii) including the case of CD already diagnosed. Therefore, the estimated prevalence of CD in the eligible study group was 2.18% (8.83/405; 95% CI, 0.8–3.7%) (see Table 2 for a comparison between the characteristics of our study and the study considered as control [16]).

The Chi-square test (see Table 3) did not show a statistically significant difference (*p*-value = 0.36) in the estimated prevalence of CD identified in our sample of children with ASD (2.18%; 95% CI: 0.8–3.7) as compared with the sample obtained from the Italian pediatric population (90.4/5705; 1.58%; 95% CI, 1.26–1.90%) with the same age range [16].

## 4. Discussion

In the current study, a large sample of children with ASD, aged 5–11 years, was tested for CD, providing new information about the controversial issue of a possible association between CD and ASD. A sample of children from the community, matched for age range and geographical origin, was used as a control group [16]. Specifically, in this study the authors screened a sample of 4570 Italian children aged 5–11 years by examining HLA genes associated with increased risk of CD and measuring total serum levels of IgA and anti-tTG IgA in children at risk for CD. Diagnoses of CD were then confirmed by the detection of anti-endomysial antibodies and the analysis of intestinal biopsies. The authors concluded that the CD prevalence in the eligible population was 1.58% (95% CI, 1.26–1.90%), calculated assuming: (i) a lack of CD cases among HLA-negative children, (ii) the same prevalence of CD among children who refused the screening (n = 1135), and (iii) the inclusion of cases in which CD was known prior to screening (n = 23).

We did not detect a higher prevalence of CD in ASD subjects (2.21%) than in the general pediatric population (1.58%). Even though this difference is not statistically significant, some considerations deserve to be discussed. First, it is worth mentioning the difference in distribution of males and females in the analyzed samples; the percentage of females was 48% in the control pediatric population [16] and only 17% in our ASD cohort, in line with the epidemiology of ASD, in which a skewed sex ratio, indicative of a greater preponderance of males over females, is constantly reported [46]. In particular, from the starting cohort of 302 males and 60 females with ASD, we finally identified five females and four males with CD, which accords with the actual prevalence of CD in young age groups, which is higher in females compared to males [47]. Therefore, the low percentage of females in our consecutive sample of ASD in comparison with the control group might have affected our overall estimates of CD, a disorder more common among females than among males. In order to reduce this bias, further investigations examining the potential association between ASD and CD should match cases with controls with respect to sex. In addition, although our overall sample size of ASD individuals was quite large, the search for a relatively uncommon condition such as CD—only eight cases of CD were detected in our sample—still makes it difficult to calculate a precise estimate of CD prevalence, with a possible increased probability of type II error.

Unlike various studies on this topic [7,26,27,33], we did not include AGA for a diagnostic purpose. Indeed, in recent years AGAs have lost much of their diagnostic value, due to the introduction of the more sensitive and specific anti-tTG test [48]. Moreover, all the positive seroprevalence cases for CD have been further investigated up to their diagnostic confirmation of CD. The diagnostic criteria for CD have changed over time: the ESPGHAN guidelines [43,44] are more stringent than previous ones, possibly contributing to the reducing rate of false-positive cases. For this reason, the prevalence of CD in our sample is presumably closer to reality, compared with previous investigations on this topic.

Despite this, considering the pooled prevalence of CD in ASD individuals identified by a recent systematic review and meta-analysis on the psychiatric manifestation of CD [49], the prevalence we detected is slightly less than twice that so identified (2.18% vs. 1.3%). It is important to emphasize that not all studies exclude subjects already on GFD, as recommended and [7] as we did, possibly causing false-negative CD diagnoses in their considered samples, and, in turn, findings of a lower prevalence of CD.

In a previous investigation on a large sample of preschoolers with ASD, we noticed that CD occurs at a frequency of 2.62%, which was statistically significantly higher than in the general pediatric population [21]. Crucially, half of ASD children with CD in that sample had a silent form of CD, with no typical CD-related symptoms or risk factors at time of serological testing. This high prevalence of the silent form of CD in ASD children could be ascribed both to its different clinical presentation, compared with the typical population, and to difficulties of assessing GI distress in children with ASD.

Our new data on an equally large sample of older ASD subjects do not seem to replicate those results. However, the percentage of ASD subjects with CD is quite like our previous study (2.18 % vs. 2.62%, respectively).

Different considerations can be made as to why this occurred. Firstly, an increase in the CD individuation in the general population was reported (the decrease of the “hidden” iceberg) and, more broadly, in the worldwide prevalence of CD [50,51]. In this context, it is important to note that the prevalence of CD identified in preschoolers with ASD [21] was compared with a CD prevalence derived from a European screening study [52] examining older serological data collection (serological CD screening performed from 2010 to 2013 vs. 1997 to 2002, respectively), possibly reflecting the different prevalence of CD based on the specific period examined. Conversely, in the current study, in addition to comparing our data with those of a similar reference population (same geographical area and age range) [16], we also referred to a similar time of sample collection (i.e., from 2014 to 2018 in the current sample, and from 2015 to 2016 in the control sample). This allows us to exclude possible bias due to differing periods of sample collection. Consequently, the current investigation results have the advantage of deriving from the comparison between two quite similar populations in terms of age, nationality, and the collection period of serological samples. Vice versa, regarding the methodology adopted, the sample we used as a comparison was screened with HLA determination as a first-level test. This choice presumably did not affect the final CD prevalence, since HLA-testing’s negative predictive value is close to 100% [53], minimizing the possibility of false negatives for CD.

As far as the CD clinical presentation in children with ASD, our results support a frequent asymptomatic form, which could be partially ascribed to the communication difficulties and the atypical sensory processing characteristic of ASD subjects, which in turn prevent the effective communication and/or the proper localization of GI symptoms [20,54,55,56]. Within this framework, we must be aware that GI symptoms can occur with sudden irritability or aggressive behavior in non-verbal children with ASD [20,56,57].

Even though this study has various strengths—namely, the large and age-homogeneous sample considered, as well as the high sensitivity of the screening algorithm used—there are some limitations to acknowledge. Firstly, we assumed that ASD children whose parents refused the CD screening had the same CD prevalence of ASD as children with CD screening. This approach may have both overestimated or underestimated the presence of CD in our sample. Still, it is motivated by using the same methodology reported in this investigation as control study, to compare our results [16].

Secondly, the presence of CD’s clinical manifestations has been evaluated through the administration of a standardized telephone interview with parents, without a particular adaptation to the clinical manifestations of CD in the ASD population. Consequently, an inaccurate assessment of symptoms, and, in particular, of GI problems, cannot be excluded, especially in younger subjects or in children with more severe autistic symptoms [58]. However, this weakness is mitigated by the high agreement between parents’ reports and gastroenterological assessments in ASD children [59,60]. Thirdly, these results were obtained in a tertiary referral center, and may therefore not be generalizable to other patient populations. Also, the compared cohorts are both composed of Italian children, but while the study sample is derived from Italy as a whole, the control sample is recruited only from two cities, i.e., Ancona and Verona.

Moreover, we cannot rule out the presence of children with fluctuating anti-tTG IgA levels: only long-term follow-up studies can determine whether these cases are actually CD-negative or are “false anti-tTG negatives” only temporarily, since they will develop full-blown CD over time [61]. Of note, the selection of ASD children aged 5 to 11 years was motivated, in this study, by the necessity of having an age-matched control group, but in this way we could have excluded ASD children under five years of age already diagnosed with CD.

Finally, we used, as a CD-prevalence control group, a pediatric sample derived from a study [16] in which no data were reported about the prevalence of ASD within the sample: therefore, we cannot exclude that, within the control sample, some ASD cases were present.

In conclusion, we did not identify an association between CD and ASD. Therefore, the usefulness of offering a population screening for CD in ASD [22] is not supported by our results. On the other hand, we must also consider that the clinical burden of unrecognized CD could be particularly important in ASD patients. Indeed, an untreated CD may worsen the pre-existing neuropsychiatric clinical picture of children with ASD by exacerbating the associated symptoms and/or by determining the onset of seemingly unexplained psychiatric manifestations [62]. For these reasons, further studies are warranted to assess the cost-benefit analysis of CD screening in asymptomatic ASD patients, and to define possible specific guidelines for the ASD condition. Concurrently, future research may benefit from more sophisticated statistical analyses, such as multivariate regression models, in order to adjust for inevitable confounding variables (e.g., family history of CD, presence of autoimmune diseases).

What is known

- Celiac disease (CD) is an immune-mediated disorder, elicited by gluten in susceptible individuals
- Studies assessing the prevalence of CD in patients with autism provide controversial results

What is new

- The study provides an updated estimate of the CD prevalence in Italian children with autism
- Our results support a frequent asymptomatic form of CD in autistic children

## Figures and Tables

**Table 1:** Biological findings and clinical characteristics of the nine ASD subjects with CD or positive CD serology.

No.	Age at First Evaluation (Years)	Sex	tTG IgA (U/mL)	Total IgA (mg/dL)	EMA	Risk Factor and Clinical Presentation	CD Diagnosis	Biopsy Type
1	5.8	F	277.0	72	+	none	tTG+HLA+	n.p.
2	9.0	F	33.0	135	+	none	tTG+SIB+	MARSH 3A
3	5.8	F	>128.0	220	+	hyporexia, poor weight gain	CL+tTG+HLA+	n.p.
4 ^a^	6.0	F	n.a.	n.a.	n.a.	poor weight gain after the first year of life	tTG+SIB+	n.a.
5	11.6	M	522.0	128	+	mild constipation, insomnia	tTG+SIB+	mod MARSH 3B
6	6.6	F	16.0	49	+	severe constipation, insomnia, irritability	CL+tTG+HLA+	refused
7	6.9	M	137.0	161	+	atopic dermatitis from birth on elbows and skin folds	CL+tTG+SIB+	MARSH 3B
8	10.3	M	55.0	58	+	none	tTG+SIB+	mod MARSH 3C
9	6.2	M	17.0	131	+	none	excluded, based on repetition of serological screening tests	

Abbreviations (alphabetic order): CD celiac disease; CL clinical features; M male; F female; tTG IgA anti-transglutaminase antibodies; EMA anti-endomysium antibodies; cut-off values for tTG IgA: positive: > 10 U/mL, borderline 7–10 U/mL, negative <7 U/mL; EMA: “+” positive result, “−” negative result, “+/−” doubt result, n.p. not performed; n.a. not applicable; HLA+ human leukocyte antigen positivity; SIB+ small intestinal biopsy positivity; MARSH Marsh classification of histologic findings; mod MARSH modified Marsh-Oberhuber classification of histologic findings. ^a^ ASD patient who had received a CD diagnosis before the hospitalization in our ASD Unit.

**Table 2:** Comparison of sample- and CD-evaluation characteristics between our study and the study considered as control (Gatti et al., 2020).

	This Study	Control Study (Gatti et al., 2020)
Type of study	Retrospective	Cross-sectional
Aim	To estimate the prevalence of CD in ASD children	To estimate the prevalence of CD in an Italian pediatric population
Evaluation period	January 2014–December 2018	May 2015–December 2016
Geographical origin	all over Italy	Ancona and Verona (Italy)
Initial sample size (n)	405	5705
CD screening not performed (n)	43	1135
CD screening performed (n)	362	4570
Previous diagnosis of CD (n)	1	23
Children with CD (n)	7	54
No symptoms of CD (n)	3	23
Supposed children with CD in the subgroup who not performed the screening (n)	1	13
Age range (years)	5–11	5–11
Number of females (%)	60 (17)	2193 (48)
Median (years)	6.58	7.88
IQR (years)	5.6–8.1	7.0–8.8
Diseases	autism spectrum disorder	not evaluated
First-level screening test		
What	Total serum IgA tTG IgA tTG IgG EMA IgA	HLA-DQ2/8 total serum IgA tTG IgA (DGP IgG)
Where	hospital setting	school
Second-level procedures		
What	screening repetition based on prior results	EMA IgA (same frozen serum)
Where	hospital setting (city of residence, Italy)	Hospital setting (G. Fracastoro hospital, Verona)
Third-level procedures		
What	SIB	SIB
Where	hospital setting (city of residence, Italy)	Hospital setting (Ancona and Verona)
Classification type for the biopsy	Marsh classification Marsh-Oberhuber classification	Marsh-Oberhuber classification

Abbreviations (alphabetic order): CD celiac disease; DGP anti-deamidated gliadin peptide antibodies; EMA anti-endomysium antibodies; HLA human leukocyte antigen; IgA immunoglobulin A; IgG immunoglobulin G; IQR interquartile range; MARSH Marsh classification of histologic findings; mod MARSH modified Marsh-Oberhuber classification of histologic findings; n number; SIB small intestinal biopsy; tTG anti-transglutaminase antibodies.

**Table 3:** Comparison between the estimated prevalence of CD in our sample and in a pediatric Italian sample matched for age.

	Number of Estimated Subjects Positive for CD Diagnosis	Number of Estimated Subjects Negative for CD Diagnosis	Total Number of Subjects (in the Eligible Study Group)	Overall Estimated Prevalence of CD	Chi-Square Statistic Value
Group 1 [Our study]	8.83	396.17	405	2.18%	n.s.
Group 2 [16]	90.40	5614.6	5705	1.58%	
Total	99.23	6010.77	6110		

Abbreviations (alphabetic order): CD: celiac disease; n.s.: not significant.

## Data Availability

The datasets used and/or analysed during the current study are available from the corresponding author on reasonable request.

## Abbreviations

a-CGH	array comparative genomic hybridization
ADOS-2	Autism Diagnostic Observation Schedule-Second Edition
AGA	anti-gliadin antibodies
anti-tTG	anti-transglutaminase
ASD	autism spectrum disorder
CD	celiac disease
EMA	anti-endomysium
ESPGHAN	European Society for Paediatric Gastroenterology, Hepatology, and Nutrition
FEIA	fluorescent enzyme immunoassay
GFD	gluten-free diet
GI	gastrointestinal
Ig	immunoglobulin
IQR	interquartile range
MHC	major histocompatibility complex
SD	standard deviation
